# Spontaneous Flexor Tendon Rupture due to Primary Distal Radioulnar Joint Osteoarthritis

**DOI:** 10.1155/2019/7604897

**Published:** 2019-04-09

**Authors:** Akira Hashimoto, Motoki Sonohata, Hideyuki Senba, Masaaki Mawatari

**Affiliations:** ^1^Department of Orthopaedic Surgery, Faculty of Medicine, Saga University, Nabeshima 5-1-1, Saga 849-8501, Japan; ^2^Department of Orthopaedic Surgery, Karatsu Red Cross Hospital, Watada 2430, Karatsu, Saga 847-8588, Japan

## Abstract

Spontaneous flexor tendon rupture is rare, occurring most commonly in the little finger or flexor pollicis longus. To the best of our knowledge, there have been no reports of spontaneous flexor tendon rupture due to primary distal radioulnar joint (DRUJ) osteoarthritis (OA). We present a case of spontaneous flexor tendon rupture in the index finger due to primary DRUJ OA in a 71-year-old female farmer. Surgical exploration confirmed that, at the wrist joint level, the flexor digitorum profundus of the index finger had undergone degeneration and complete rupture. The flexor digitorum superficialis of the index finger was elongated and thinned. A bony spur toward the volar side was covered with synovial fluid from a pinhole-sized perforation of the capsule. The combination of direct friction from the DRUJ spur and the matrix metalloproteinases in the synovial fluid from the perforation of the DRUJ capsule may have caused the spontaneous flexor tendon rupture. Palmar-side symptoms associated with DRUJ OA should be carefully examined because of the risk of spontaneous flexor tendon rupture.

## 1. Introduction

Spontaneous flexor tendon rupture is relatively uncommon and is usually caused by trauma, inflammatory disease, steroids (injection or oral therapy), or surgical complications from plate, and carpal bone and joint disorders [1, 2]. There have been reports of unusual causes of spontaneous flexor tendon rupture, scaphoid nonunion, hamate hook nonunion, Kienböck's disease, dorsal intercalated segment instability, and pisotriquetral osteoarthritis (OA) [3–7]. Although there are reports of extensor tendon rupture associated with primary distal radioulnar joint (DRUJ) OA [8, 9], to the best of our knowledge, there have been no reports of spontaneous flexor tendon rupture due to primary DRUJ OA. We report a case of spontaneous flexor tendon rupture in an index finger due to primary DRUJ OA.

The study protocol adhered to the ethical guidelines of the 1975 Declaration of Helsinki, and the study was approved by the institutional review broad of our institute. The patient was informed that this case study would be submitted for publication, and she provided informed consent.

## 2. Case Presentation

**Figure 1:**
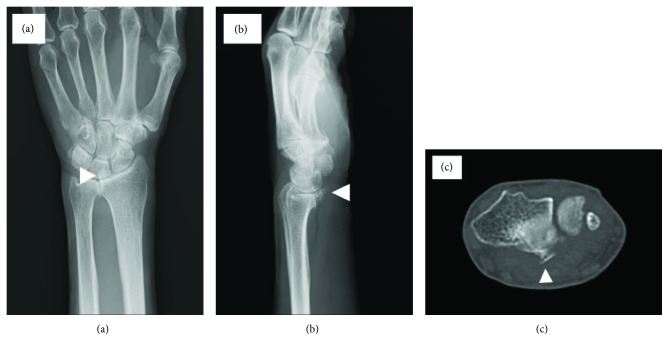
Clinical examination. (a) Bony spur on the anterior-posterior plain radiograph of the wrist joint (white arrowhead). (b) Bony spur on the lateral plain radiograph of the wrist joint (white arrowhead). (c) Bony spur on computed tomography (white arrowhead).

A 71-year-old woman was prescribed analgesics at another orthopedic clinic because of pain and swelling in the right carpal area. Half a month later, she could not flex the index finger of her right hand. She had no past history of trauma, carpal bone and joint disorders, or inflammatory disease and had not taken any steroid injection recently. She has been a farmer for a long time. On clinical examination, she was not able to actively flex the distal interphalangeal joint of her index finger. The proximal interphalangeal joint could be flexed to 40°. The anterior-posterior and lateral plain radiographs showed a bony spur arising from the volar ulnar aspect of the distal radius (Figures 1(a) and 1(b)). Computed tomography revealed that the bony spur from the radius was a part of DRUJ OA (Figure 1(c)).

**Figure 2:**
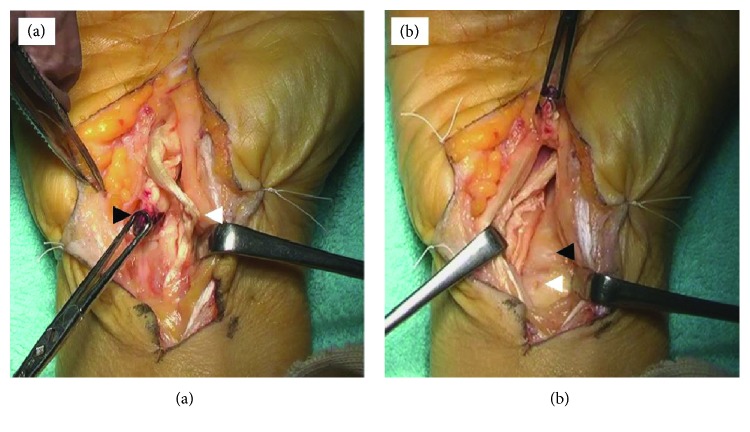
Intraoperative findings. (a) Rupture of the flexor digitorum profundus of the index finger (black arrowhead). Degeneration of the flexor digitorum superficialis of the index finger (white arrowhead). (b) A pinhole-sized perforation of the distal radioulnar joint capsule (white arrowhead). The bony spur covered with a joint capsule (black arrowhead).

During surgery, under general anesthesia and using tourniquet control, a zig-zag incision was made at the level of the DRUJ on the palmar side. Surgical exploration confirmed that at the wrist joint level, the flexor digitorum profundus (FDP) of the index finger had undergone degeneration and complete rupture. The flexor digitorum superficialis (FDS) of the index finger was elongated and thinned. The FDP of the middle finger had undergone slight degeneration; however, tension of the FDP of the middle finger was normal (Figure 2(a)). The bony spur toward the volar side was covered with a joint capsule (Figure 2(b)). The volar capsule of the DRUJ had a pinhole-sized perforation (Figure 2(b)). There was synovial fluid from the pinhole-sized perforation (Figure 2(b)). Resection of the bony spur and the DRUJ capsule repair were performed. Then we performed single-stage reconstruction of the FDP of the index finger with a right palmaris longus bridge graft using interlacing 4-0 nylon sutures.

## 3. Discussion

DRUJ OA occurs as the result of a variety of mechanisms, including primary lesions, inflammatory arthritis (particularly rheumatoid arthritis), posttraumatic causes (a malunited Colles' fracture of the distal radius, Galeazzi fracture-dislocation), and congenital or developmental abnormalities of the joint surfaces (spondylometaphyseal dysplasias, ulnar impaction syndrome) [10, 11]. The frequency of primary DRUJ OA was 12.3% in a cross-sectional study [11].

The most common complication of DRUJ OA is extensor tendon rupture [12]. Nevertheless, there are reports of flexor tendon rupture due to DRUJ OA after Galeazzi fracture-dislocation [13, 14]; however, there have been no reports of flexor tendon rupture due to primary DRUJ OA. The flexor tendons are more difficult to rupture than the extensor tendons, for the following reasons: (1) the flexor tendons are protected from direct injury by the pronator quadratus muscle [15], (2) the flexor tendons are not restricted and are not located in the radial compartment, as are the extensor tendons [15, 16], and (3) the flexor tendons are strong components of the musculotendinous junction and are stretched by a hypertension mechanism [15, 17].

Most reports of spontaneous extensor tendon rupture due to DRUJ OA may have involved perforation of the DRUJ capsule [8, 11, 12, 18]. Only Tanaka et al. reported a case of extensor tendon rupture due to DRUJ OA [9]. There have also been reports on the extensor tendon rupture due to DRUJ OA without perforation of the capsule of the DRUJ. Ohara et al. reported that one of the reasons for flexor tendon rupture with DRUJ OA after Galeazzi fracture-dislocation was capsule perforation [14]. In the past reports, it has been reported that matrix metalloproteinase- (MMP-) 1, MMP-3, MMP-8, and MMP-13 are expressed in synovial fibroblasts of OA [14, 19–21]. MMP-1, MMP-8, and MMP-13 also reportedly degrade type I collagen that is abundant in tendons [13, 22]. In our case as well, a pinhole-sized perforation of the DRUJ capsule was recognized and joint fluid containing MMP leaked out, which led to further degeneration of the flexor tendons. We believe that the combination of direct friction from the DRUJ spur and the MMP that degraded type I collagen resulted in spontaneous flexor tendon rupture.

In conclusion, DRUJ OA is a rare cause of flexor tendon rupture and attrition. Attention should be paid when evaluating a patient with palmar-side symptoms of the wrist joint with DRUJ OA because of a risk of spontaneous flexor tendon rupture.
